# Sunny Rooms for Health

**Published:** 1891-05

**Authors:** 


### SUNNY ROOMS FOR HEALTH.

The rooms occupied by children should be made bright, light, and pleasant. It is seldom thought of as much as it should be, how essential to the health of children plenty of light—especially sunlight—is. One reason why poor people's children thrive in the face of most adverse surroundings is that they are nearly all day out of doors in the full light of day and in the air. Keeping children excluded from sunlight and putting them in dark, gloomy rooms, is similar to caging a young bird and keeping it always in the shade; it will soon droop and lose all brightness, becoming dull and songless. Some children look pale and delicate, although surrounded with every comfort—nay, luxury—well fed, well looked after, and the real cause is often want of light—want of sunlight—and want of cheerfulness in the people and in the rooms they inhabited.

